# Bioinformatics analysis of differentially expressed genes in subchondral bone in early experimental osteoarthritis using microarray data

**DOI:** 10.1186/s13018-020-01839-8

**Published:** 2020-08-08

**Authors:** Zhao Wang, Yong Ji, Hong-wei Bao

**Affiliations:** 1 Department of Orthopaedics, Jingjiang People’s Hospital, No. 28, Zhongzhou Road, Jingjiang, Taizhou, 214500 Jiangsu Province China; 2 Department of General Surgery, Jingjiang People’s Hospital, No. 28, Zhongzhou Road, Jingjiang, Taizhou, 214500 Jiangsu Province China

**Keywords:** Osteoarthritis, Differentially expressed genes, Bioinformatics analysis, Gene ontology

## Abstract

**Background:**

Osteoarthritis (OA) is the most common arthritic disease in humans, affecting the majority of individuals over 65 years of age. The aim of this study is to identify the gene expression profile specific to subchondral bone in OA by comparing the different expression profiles in experimental and sham-operation groups.

**Methods:**

Gene expression profile GSE30322 was downloaded from the Gene Expression Omnibus (GEO) database. Differentially expressed genes (DEGs) were obtained by limma package. And Database for Annotation, Visualization and Integrated Discovery (DAVID) databases were further used to identify the potential gene ontology (GO) and Kyoto Encyclopedia of Genes and Genomes (KEGG) pathways. Furthermore, a protein–protein interaction (PPI) network was constructed and significant modules were extracted.

**Results:**

Totally, 588 DEGs were identified including 199 upregulated DEGs and 389 downregulated DEGs screened in OA and sham-operation. GO showed that DEGs were significantly enhanced for ribosomal subunit export from nucleus and molting cycle. KEGG pathway analysis revealed that target genes were enriched in thiamine metabolism.

**Conclusion:**

These key candidate DEGs that affect the progression of OA, and these genes might serve as potential therapeutic targets for OA.

## Introduction

Osteoarthritis (OA) is a degenerative disease characterized by the gradual degeneration of articular cartilage, joint stiffness, and loss of function [1]. It was reported that over 27 million adults are affected by OA in the USA [2]. OA is a complex pathophysiological process involving inflammation, subchondral bone modification, and osteophyte formation. Subchondral bone alteration present to the cartilage degeneration and thus more studies should be focused on the subchondral bone alteration.

Subchondral bone consists tripartite: subchondral bone plate, trabecular bone, and bone marrow space [3]. It has been stated that most of the OA patients accompanied by the alterations of the subchondral bone [4]. Subchondral bone could transport nutrients or cytokines to the overlying cartilage. Meanwhile, subchondral bone cells contacted with chondrocyte and thus influence cartilage metabolism. A better understanding of the early molecular mechanism changes of subchondral bone in vivo may contribute to elucidating the pathogenesis of OA. Therefore, it is crucial to explore the differentially expressed genes (DEGs) in vivo and thus we could revealed new targets for OA [5].

Microarray technology has been used to obtain information on the genetic alteration that occurs during many diseases [6, 7]. Here, we downloaded the gene expression profile GSE30322 from the Gene Expression Omnibus database (GEO), including gene expression data for subchondral bone samples from five medial meniscectomy and medial collateral ligament transection group and five sham-operated group. Based upon this research, identifying DEGs and enriching their functions and signaling pathways may help reveal potential targets of early OA.

## Materials and methods

### Gene expression microarray data

The gene expression profile GSE30322 (https://www.ncbi.nlm.nih.gov/geo/query/acc.cgi?acc=GSE30322) was downloaded from the Gene Expression Omnibus (GEO, www.ncbi.nlm.nih.gov/geo/). GSE30322 was based on Agilent-014879 Whole Rat Genome Microarray 4x44K G4131F (Probe Name version) platform. GSE30322 dataset contained ten samples, including five bone 4 weeks post-surgery samples (E-group), and five sham-operated group (S-group) 4 weeks post-surgery samples.

### DEGs in E-group and intact S-group samples

The raw data files were downloaded and then python scripts for matrix transformation were used. The analysis was carried out using Limma package from Bioconductor project. In this study, genes with *P* < .05 and [log fold change (FC)] > 2 were defined as DEGs. The DEGs data were then processed by R software (pheatmap package) to draw a heatmap and volcano plot.

### GO and KEGG analysis of DEGs

Target genes list were submitted to the DAVID 6.8 (https://david.ncifcrf.gov/) to analyze candidate DEG functions and Kyoto Encyclopedia of Genes and Genomes (KEGG) of the overlapping genes. DEG functions, also named as Gene ontology (GO), mainly including biological process (BP), molecular function (MF), and cellular component (CC). *P* value less than 0.05 was considered as cut-off criterion [8–10].

### Protein-protein interaction (PPI)

We used the online database STRING (Search Tool for the Retrieval of Interacting Genes, https://string-db.org/) to better illustrate the potential interactive relationships among the DEGs [11]. Then, the Cytoscape software was utilized for analyzing the interactions with a combined score > 0.4 (http://www.cytoscape.org/). Finally, the plug-in Molecular Complex Detection (MCODE) was used to filter the significant modules from the PPI network for the selection of hub genes (degree cut-off = 2, node score cut-off = 0.2, *k*-core = 2, and max. depth = 100) [12].

## Results

### Identification of DEGs

**Fig. 1:**
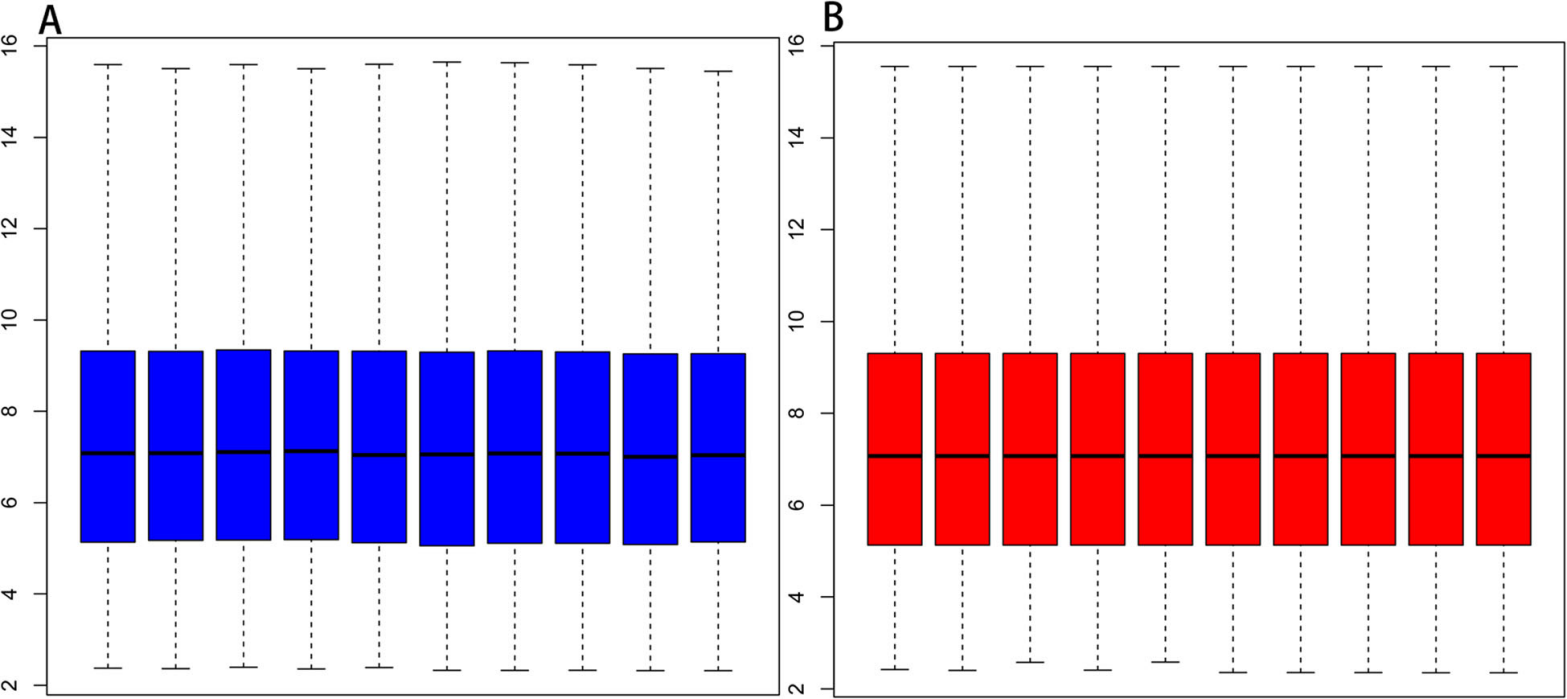
Box plot for the sample data after normalization

**Fig. 2:**
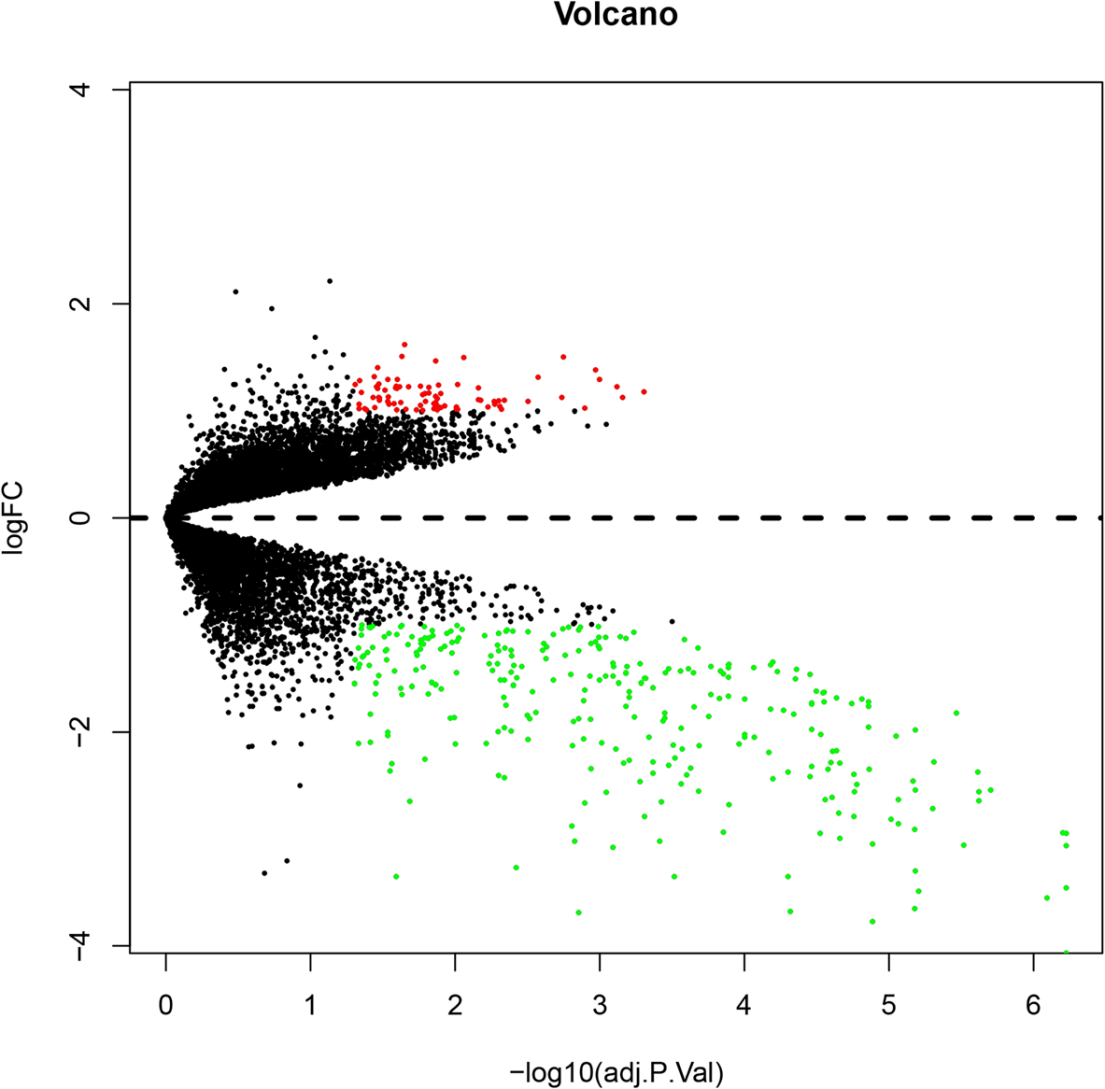
Volcano plot of the different genes in E-group and S-group

**Fig. 3:**
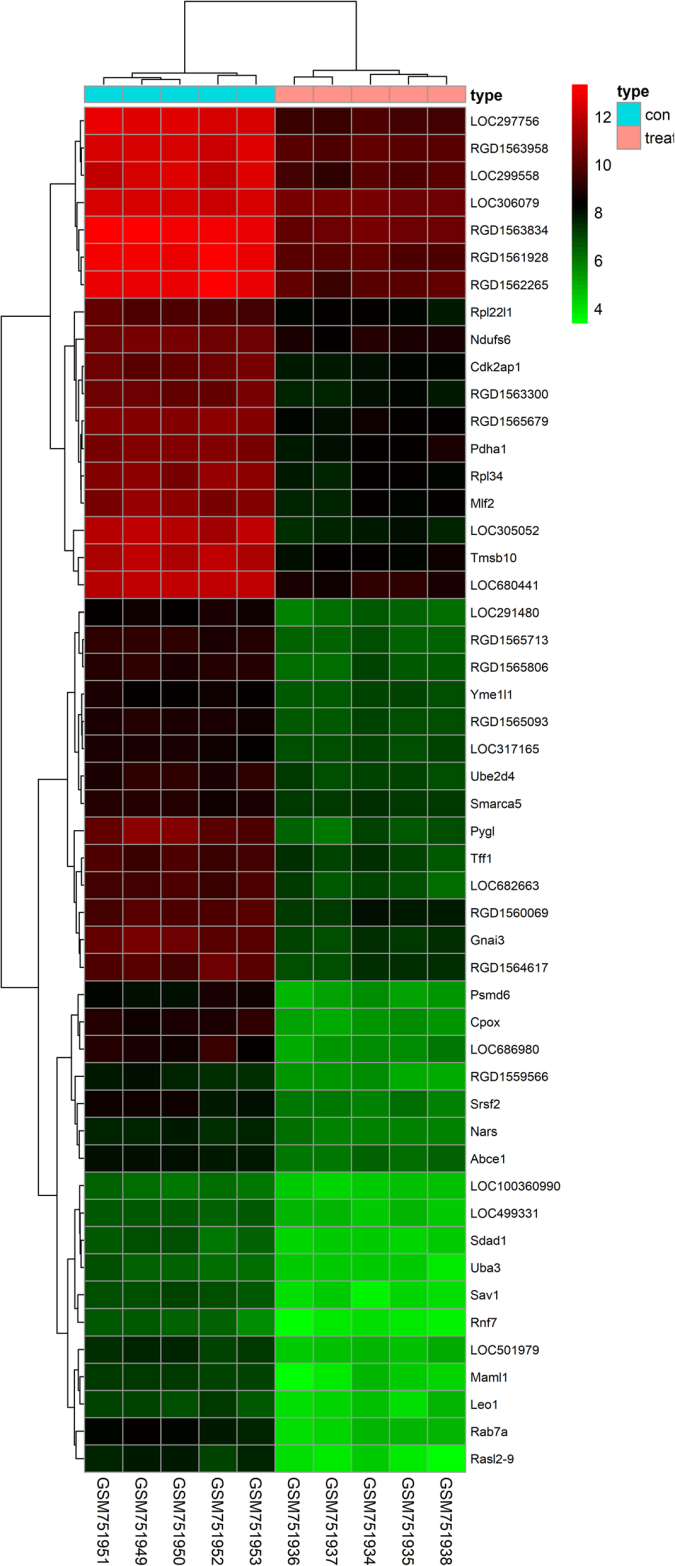
Heat map of the top fifty different genes in E-group and S-group

After analyzing, differentially expression gene profiles were obtained. Totally, 588 DEGs were identified including 199 upregulated DEGs and 389 downregulated DEGs screened in OA and sham-operation. Top 10 up-DEGs and down-DEGs were listed in Table 1 and Table 2, respectively. A box plot of the sample data is provided in Fig. 1. Volcano plot of the different genes can be obtained in Fig. 2. Moreover, we provided heatmap of the top 50 different genes between E-group and S-group (Fig. 3).

**Table 1:** The top 10 upregulated DEGs in early experimental osteoarthritis with *P* value < 0.05

Genesymbol	logFC	*P* value
Rhox5	2.271332	0.008302
Bex1	2.035975	0.040834
RGD1309085	1.726136	0.012291
Nsg1	1.658621	0.001075
Klhdc5	1.63377	0.007725
Trpc4	1.609058	0.003723
Klrd1	1.606762	0.00939
Fgfbp3	1.604729	0.000907
Gzma	1.588101	0.011649
Nr4a3	1.550933	1.16E-05

**Table 2:** The top 10 downregulated DEGs in early experimental osteoarthritis with *P* value < 0.05

Genesymbol	logFC	*P* value
Ric8a	− 4.51046	3.57E−06
Fth1	− 4.09365	2.18E−05
LOC305052	− 4.0216	1.25E−10
Pygl	− 3.73589	3.01E−08
Cks2	− 3.61584	3.06E−07
Usp4	− 3.60799	2.21E−05
Rasl2-9	− 3.57891	1.54E−08
Cpox	− 3.50355	3.86E−10
Rab7a	− 3.43118	1.07E−08
Tmsb10	− 3.40738	1.24E−10

### GO term enrichment analysis of DEGs

**Fig. 4:**
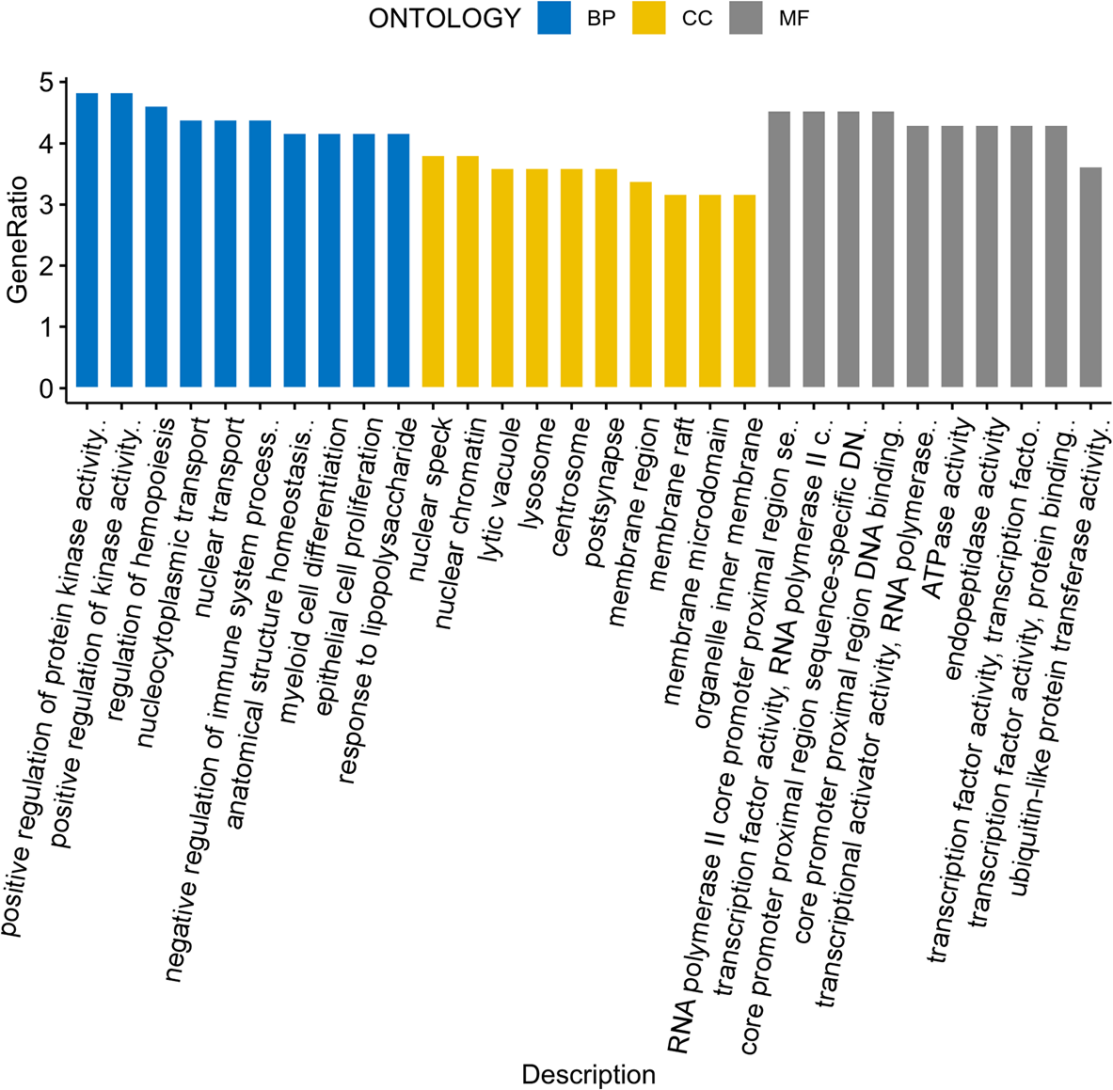
Gene ontology analysis of differentially expressed genes in E-group and S-group

Gene Ontology (GO) showed that up-DEGs were significantly enhanced for ribosomal subunit export from nucleus, ribosome localization, regulation of hemopoiesis, negative regulation of hemopoiesis, and rRNA-containing ribonucleoprotein complex export from nucleus. Downregulated DEGs were enriched for the molting cycle, hair cycle, molting cycle process, hair cycle process, and the skin epidermis development (Table 3 and Fig. 4).

**Table 3:** Gene ontology analysis of differentially expressed genes in E-group and S-group according to BP, CC and MF

ONTOLOGY	Description	*P* value	*P* adjust	gname
BP	Ribosomal subunit export from nucleus	0.000659	0.43601	RASL2-9/SDAD1/ZFP593/RPS15
BP	Ribosome localization	0.000659	0.43601	RASL2-9/SDAD1/ZFP593/RPS15
BP	Regulation of hemopoiesis	0.000698	0.43601	ADRM1/LEO1/CSF1/HMGB2/CALCA/RARA/ITPKB/NFE2L2/MEIS2/ERBB2/IHH/GPR171/INHBA/TNFSF9/PPP2R3C/MIXL1/HIST2H4/GNAS/IL1F8/ZAP70/RGD1309676
BP	Negative regulation of hemopoiesis	0.001031	0.43601	LEO1/CALCA/RARA/ITPKB/NFE2L2/MEIS2/ERBB2/IHH/GPR171/MIXL1/HIST2H4
BP	rRNA-containing ribonucleoprotein complex export from nucleus	0.001063	0.43601	RASL2-9/SDAD1/ZFP593/RPS15
BP	Hair follicle development	0.001079	0.43601	SAV1/VANGL2/LHX2/GAL/INHBA/RBPJ/GNAS/LGR4/PRSS8
BP	Molting cycle	0.001083	0.43601	SAV1/VANGL2/LHX2/GAL/INHBA/RBPJ/GNAS/LGR4/ARNTL/PRSS8
BP	Hair cycle	0.001083	0.43601	SAV1/VANGL2/LHX2/GAL/INHBA/RBPJ/GNAS/LGR4/ARNTL/PRSS8
BP	Molting cycle process	0.001245	0.43601	SAV1/VANGL2/LHX2/GAL/INHBA/RBPJ/GNAS/LGR4/PRSS8
BP	Hair cycle process	0.001245	0.43601	SAV1/VANGL2/LHX2/GAL/INHBA/RBPJ/GNAS/LGR4/PRSS8
CC	Cytosolic small ribosomal subunit	0.000355	0.163784	LOC297756/RPS7/RPS26/RPS27A/RGD1565117/RGD1562381/RPS15/RGD1559808
CC	Nuclear speck	0.002082	0.276812	SURF2/EP400/SRSF2/PACSIN2/GLYR1/BASP1/KIF22/SNRPB2/MORF4L1/SMC4/CKAP4/TCF3/NR3C1/HSPB3/HSPA1B/CDK12/CNOT7/EAF2
CC	Blood microparticle	0.002809	0.276812	ACTC1/CPN2/HBE1/PRSS1/HSPA1B/ACTG2/PROS1/HSPA1L/SERPINF2
CC	Small ribosomal subunit	0.002991	0.276812	LOC297756/RPS7/RPS26/RPS27A/RGD1565117/RGD1562381/RPS15/RGD1559808
CC	Cytosolic ribosome	0.003002	0.276812	LOC297756/RPL34/RPS7/LOC306079/RPS26/SURF6/RPS27A/RGD1565117/RGD1562381/RPS15/RGD1559808
CC	Autophagosome membrane	0.003616	0.27785	RAB7A/LAMP2/STX17/PRKD1
CC	Nuclear chromatin	0.004689	0.308812	EP400/ARID1A/RBMXRTL/SFR1/HMGB2/RARA/CBX5/SMARCA5/MORF4L1/PSIP1/TCF3/RUNX2/MIXL1/ETV3/HIST2H4/HIST1H1A/MXD1/HIST1H1B
CC	Autophagosome	0.016393	0.669112	FTH1/RAB7A/LAMP2/STX17/PRKD1/ATG12
CC	DNA packaging complex	0.016574	0.669112	HIST2H2BE/GLYR1/SMC4/TCF3/HIST2H4/HIST1H1A/HIST1H1B
CC	Lateral plasma membrane	0.016808	0.669112	VANGL2/NSG1/PKD1/FGF13/GJB2
MF	Repressing transcription factor binding	0.00545	0.759982	CBX5/TCF3/RUNX2/RBPJ/MIXL1/ARNTL
MF	Structural constituent of ribosome	0.006363	0.759982	LOC297756/RPL34/RGD1564617/RPS7/RGD1562397/LOC306079/RPS26/RPS27A/RGD1565117/LOC100360679/RGD1562381/RPS15/MRPL3/RGD1559808
MF	RNA polymerase II core promoter proximal region sequence-specific DNA binding	0.00642	0.759982	NR4A3/LHX2/TCF3/NR3C1/ZFP384/ALS2CR8/MEIS2/RUNX2/GMEB2/SOX6/RBPJ/HIVEP2/NKX6-1/MIXL1/PITX3/BCL11B/FOXS1/PAX1/MXD1/CRX
MF	Transcription factor activity, RNA polymerase II core promoter proximal region sequence-specific binding	0.008708	0.759982	NR4A3/LHX2/TCF3/NR3C1/ZFP384/ALS2CR8/MEIS2/RUNX2/SOX6/RBPJ/HIVEP2/NKX6-1/MIXL1/PITX3/BCL11B/FOXS1/PAX1/MXD1/ARNTL/CRX
MF	Core promoter proximal region sequence-specific DNA binding	0.008925	0.759982	NR4A3/LHX2/TCF3/NR3C1/ZFP384/ALS2CR8/MEIS2/RUNX2/GMEB2/SOX6/RBPJ/HIVEP2/NKX6-1/MIXL1/PITX3/BCL11B/FOXS1/PAX1/MXD1/CRX
MF	Core promoter proximal region DNA binding	0.009372	0.759982	NR4A3/LHX2/TCF3/NR3C1/ZFP384/ALS2CR8/MEIS2/RUNX2/GMEB2/SOX6/RBPJ/HIVEP2/NKX6-1/MIXL1/PITX3/BCL11B/FOXS1/PAX1/MXD1/CRX
MF	Manganese ion binding	0.011064	0.759982	GLUL/XPNPEP1/IMPA1/ARG1/TDP2
MF	Transcriptional activator activity, RNA polymerase II core promoter proximal region sequence-specific binding	0.011141	0.759982	NR4A3/LHX2/TCF3/NR3C1/ZFP384/ALS2CR8/MEIS2/RUNX2/RBPJ/HIVEP2/MIXL1/PITX3/BCL11B/PAX1/CRX
MF	Histone binding	0.011785	0.759982	CKS2/GLYR1/HMGB2/PHF1/CBX5/SMARCA5/USP15/ING1/ATAD2/HIST2H4/RAG2
MF	Methylated histone binding	0.016167	0.759982	GLYR1/PHF1/CBX5/ING1/RAG2

### KEGG pathway analysis of DEGs

**Fig. 5:**
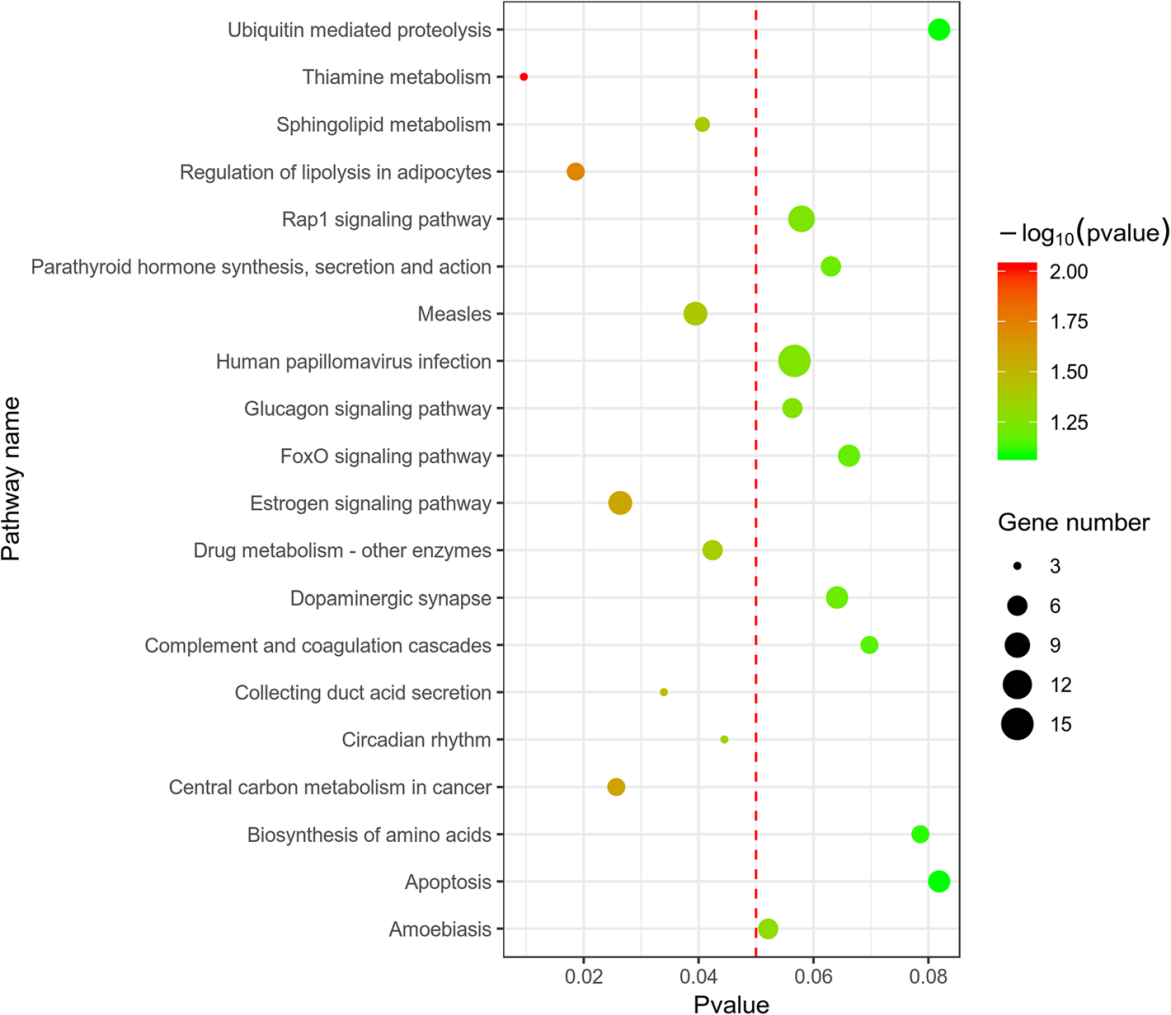
The results of KEGG pathways enrichment analysis for DEGs based on clusterProfiler

The result of KEGG pathway analysis revealed that target genes were enriched in thiamine metabolism, regulation of lipolysis in adipocytes, central carbon metabolism in cancer, estrogen signaling pathway, collecting duct acid secretion, Rap1 signaling pathway, measles, sphingolipid metabolism, drug metabolism-other enzymes, and circadian rhythm. These key pathways were showed in Table 4 and Fig. 5.

**Table 4:** KEGG pathway enrichment analysis of differentially expressed genes in E-group and S-group

ID	Description	pvalue	p.adjust	gname
rno00730	Thiamine metabolism	0.009585	0.866414	ALPL/AKP3/AK1
rno04923	Regulation of lipolysis in adipocytes	0.018613	0.866414	GNAI3/PIK3CD/PLA2G16/AQP7/GNAS
rno05230	Central carbon metabolism in cancer	0.025683	0.866414	PDHA1/HK1/PIK3CD/PFKP/ERBB2
rno04915	Estrogen signaling pathway	0.026369	0.866414	GNAI3/TFF1/PIK3CD/RARA/HSPA1B/ITPR1/GNAS/HSPA1L
rno04966	Collecting duct acid secretion	0.033955	0.866414	ATP6V1G3/ATP6V1E1/CLCNKB
rno05162	Measles	0.039454	0.866414	PIK3CD/IFIH1/EIF3H/RAB9A/FASLG/HSPA1B/CCND3/HSPA1L
rno00600	Sphingolipid metabolism	0.040658	0.866414	B4GALT6/PPAP2B/ACER2/CERS1
rno00983	Drug metabolism—other enzymes	0.042438	0.866414	ITPA/CES2A/UGT2B5/GUSB/GSTA5/NAT2
rno04710	Circadian rhythm	0.044488	0.866414	PRKAB2/ARNTL/PER3

### Interaction network of DEGs and core genes in the interaction network

**Fig. 6:**
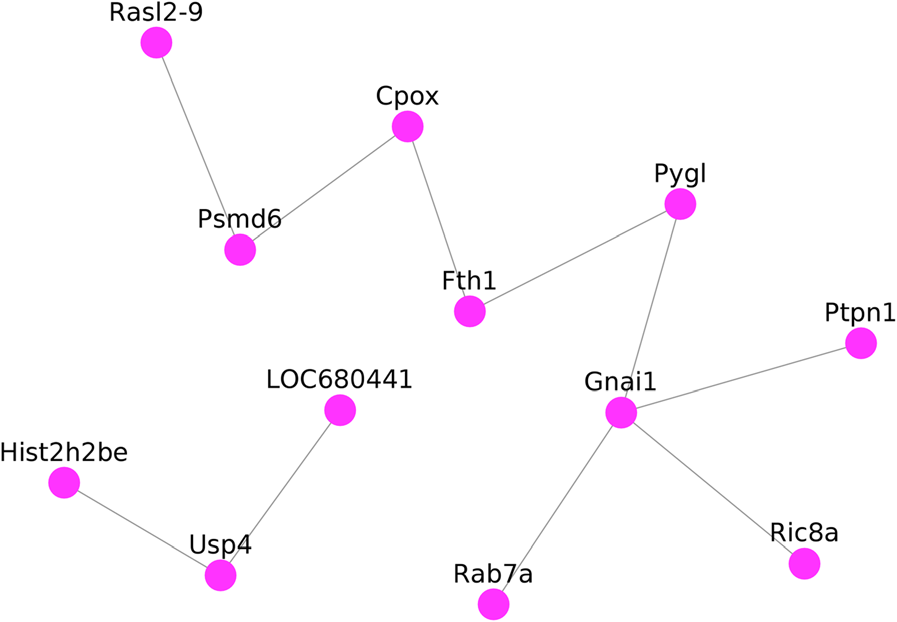
Protein-protein interaction network of differentially expressed genes. Red nodes represent upregulated genes. Blue nodes represent downregulated genes. Green nodes represent the top 10 genes. Nodes > 10 was set as cut-off criteria

**Fig. 7:**
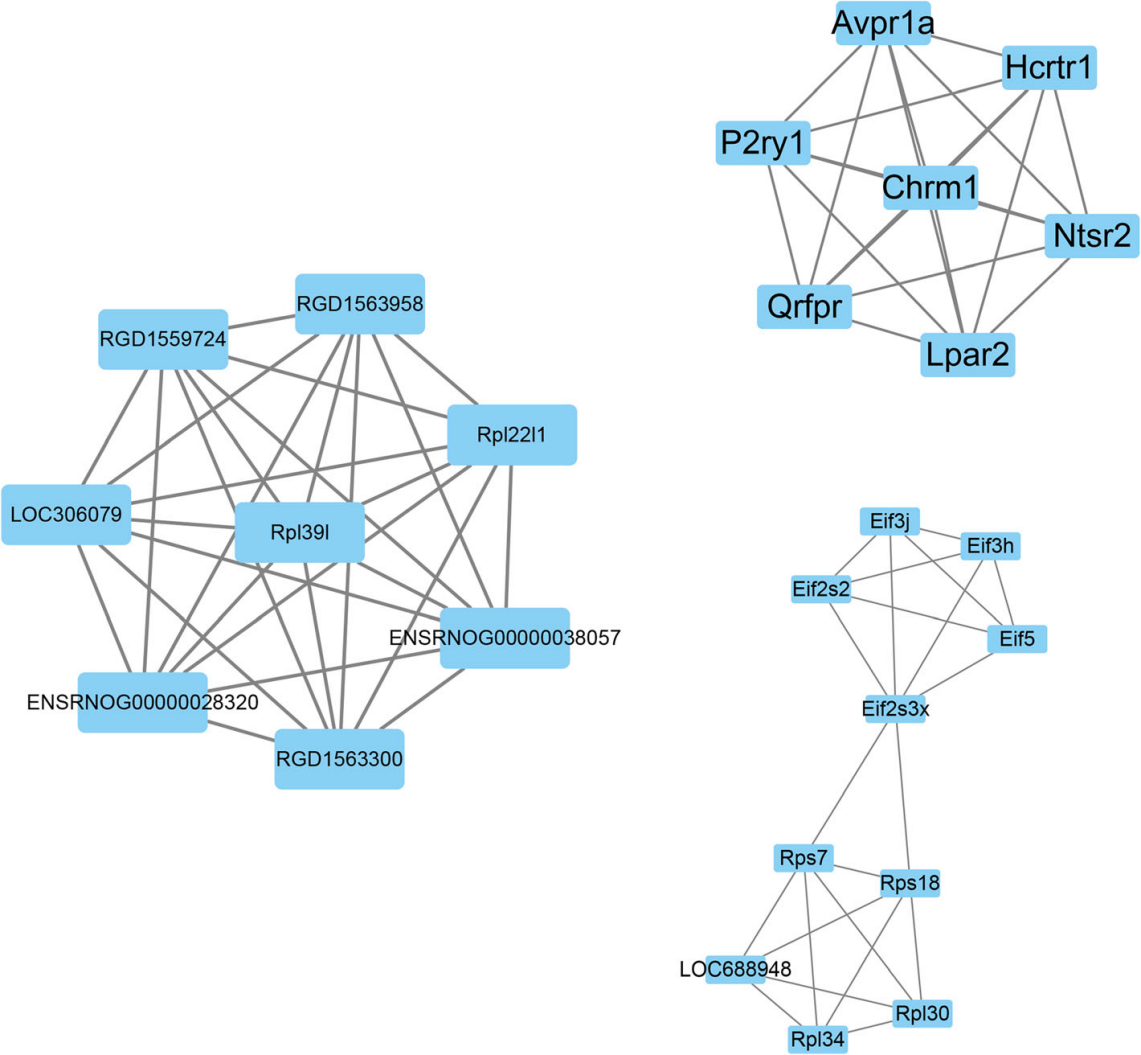
The top 3 modules from the gene–gene interaction network. The squares represent the differentially expressed genes (DEGs) in modules, and the lines show the interaction between the DEGs

Using data from the Cytoscape and STRING databases, the 10 hub nodes with the greatest degree of network connection were determined. The top 10 hub genes identified were RASL2-9, PSMD6, CPOX, FTH1, PYGL, GNAI1, PTPN1, RIC8A, RAB7A, LOC680441, USP4, and HIST2H2BE (Fig. 6 and Table 3). We listed top three MCODE results in Fig. 7.

## Discussion

Subchondral bone remodeling is regulated by the bone resorption and bone formation, which mainly regulated by osteoclast and osteoblast respectively. Despite advancements in the understanding of the mechanism of OA, an effective method for accelerating the process remains to be identified. In this analysis, we found that 588 DEGs between E-group and S-group.

Bone remodeling is regulated by the balanced processes of osteoclast-mediated bone resorption and osteoblast-mediated bone formation [13, 14]. Disequilibrium of this balance leads to dysregulated bone tissue remodeling and can result in excessive bone loss or extra bone formation and consequent skeletal disease [15]. We identified DEGs that play roles in osteoclast and osteoblast differentiation and function in the early stages of OA in this model. Previous studies identified that an altered phenotype of subchondral osteoblasts and osteoclasts contribute to OA progress. Lamas et al. [16] reported that COL10A1 downregulation seems to have a role in the establishment of a defective and/or unstable subchondral cartilage matrix in OA disease. Ren et al. [17] used gene expression profile GSE103416 to identify the different expression genes and potential pathways. Results show that Gna13/cGMP-PKG signaling pathway was identified as a potential research target for therapy and for further understanding the development of OA.

We further performed Kegg pathways to identify the potential pathways that involving in the progress of OA. We found that Rap1 signaling pathway was the most obvious different signaling pathways. For Rap1 signaling pathway, we found nine DEGs (GNAI3/RAPGEF5/CSF1/PIK3CD/MK1/LPAR2/P2RY1/GNAS/PRKD1/RASSF5). Another potential pathway was estrogen signaling pathway. Ren et al. [17] used gene expression profile GSE103416 and found that Gna13/cGMP-PKG signaling pathway was identified as a potential research target for therapy and for further understanding the development of OA. Feng et al. [18] revealed that PDGFRB, IFNG, EGR1, FASLG, and H3F3B may be the potential targets for OA diagnosis and treatment. Liang Y al [19]. found that estrogen deficiency is closely related to the development of menopausal arthritis including OA. Estrogen acts via ER and miR-140 to inhibit the catabolic activity of proteases within the chondrocyte extracellular matrix.

We also found that Rap1 signaling pathway participated into the pathological process of OA. Zhang et al. [20] performed a gene expression analyses of subchondral bone in early experimental osteoarthritis by microarray. Results found that Alp, Igf1, Tgf β1, Postn, Mmp3, Tnfsf11, Acp5, Bmp5, Aspn, and Ihh genes that involved in the pathological process of OA, and they also performed PCR to identify these DEGs in the OA and normal cartilage. Kovács et al. [21] also revealed that the Wnt and the OPG-RANKL-RANK signaling systems, as key mediators, interact in subchondral bone remodeling in OA development, which indicated that subchondral bone remodeling also affects OA. Zhou et al. [22] revealed that matrix metalloproteinase (MMP)1, MMP3, MMP13, and prostaglandin-endoperoxide synthase 2 (PTGS2) are associated with human developmental chondrogenesis.

## Conclusion

In summary, totally, 588 DEGs were identified including 199 upregulated DEGs and 389 downregulated DEGs screened in OA and sham-operation. We identified 12 core genes, including RASL2-9, PSMD6, CPOX, FTH1, PYGL, GNAI1, PTPN1, RIC8A, RAB7A, LOC680441, USP4, and HIST2H2BE. Additional experimental studies will be needed to validate our findings.

## Data Availability

The data was freely downloaded from the public GEO database.

## Abbreviations

OA: Osteoarthritis
DAVID: Database for Annotation, Visualization and Integrated Discovery
DEGs: Differentially expressed genes
GO: Gene ontology
KEGG: Kyoto Encyclopedia of Genes and Genomes
PPI: Protein–protein interaction
GEO: Gene Expression Omnibus
FC: Fold change
BP: Biological process
MF: Molecular function
CC: Cellular component
STRING: Search Tool for the Retrieval of Interacting Genes
MCODE: Molecular Complex Detection
